# RNA methylation-related genes of m6A, m5C, and m1A predict prognosis and immunotherapy response in cervical cancer

**DOI:** 10.1080/07853890.2023.2190618

**Published:** 2023-04-12

**Authors:** Yan Wang, Yiwen Mao, Caizhi Wang, Xuefeng Jiang, Qionglan Tang, Lingling Wang, Jialei Zhu, Mengqiu Zhao

**Affiliations:** a Department of Obstetrics and Gynecology, The First Affiliated Hospital of Jinan University, Guangzhou, China; b Department of Obstetrics and Gynecology, The First Affiliated Hospital of Bengbu Medical College, Bengbu, China; c Department of Surgery, Huaiyuan County Hospital of Traditional Chinese Medicine, Bengbu, China; d Department of Pathology, Sun Yat-sen Memorial Hospital, Sun Yat-sen University, Guangzhou, People’s Republic of China

**Keywords:** Cervical cancer, m6A, m5C, m1A, immune infiltration, immunosuppressive therapy, potential drug therapy

## Abstract

**Purpose:**

To investigate the prognostic value of N6-methyladenosine (m6A)-, 5-methylcytosine (m5C)-, and N1-methyladenosine (m1A)-related genes in cervical cancer (CESC) and predicting immunotherapy response.

**Methods:**

We downloaded cervical cancer mRNA expression profiles, clinical data, and m6A, m5C, m1A-related genes from public databases, and subjected them to serial bioinformatics analysis and clinical sample validation.

**Results:**

Differential analysis revealed 106 methylation-related differential genes (MEDs), including 44 differentially downregulated and 62 upregulated genes. We then obtained methylation models containing 10 genes by univariate and multifactorial COX analysis. High risk genes with HR > 1 include IQGAP3, PTBP1, STAC3, CUX1, SLC2A1, and CA2, and low risk genes with HR < 1 include IGBP1, DUOX1, CHAF1A, and STAC3. We verified the accuracy of the model from inside TCGA and outside GSE39001 (AUC = 0.729). K-M analysis showed shorter survival times in the High-risk group, and Immunocytic infiltration analysis showed model genes closely associated with six immune cells. The high-risk group may benefit more effectively from immunosuppressive therapy, especially anti-CTLA-4 therapy (*p* < .05). We also screened nine drugs for potential treatment and verified the expression of three key genes SLC2A1, CUX1, and CA2 using immunohistochemistry and RT-qPCR experiments with clinical samples.

**Conclusion:**

We identified a prognostic model using m6A/m5C/m1A-related genes in cervical cancer, which can predict survival time and correlate with immune cell infiltration. Additionally, anti-CTLA-4 may be used as an immunotherapeutic agent for cervical cancer.

**KEY MESSAGES**
- Cervical cancer still has a high mortality rate, we aim to establish a strong prognostic index and new treatment goals for improving patient survival.
- The role of three types of RNA methylation modifications, m6A, m5C, and m1A, in cervical cancer, remains unknown. Therefore, it is essential to explore the potential molecular mechanisms of m6A, m5C, and m1A methylation regulation in cervical cancer.
- We also screened nine drugs for potential treatment and anti-CTLA-4 may be used as an immunotherapeutic agent for cervical cancer. We verified the expression of three key genes SLC2A1, CUX1, and CA2

## Introduction

The high incidence of cervical cancer, which ranks first among the three major female gynaecological tumours, is closely related to persistent HPV infection, disturbance of the vaginal microecological environment, and impure sexual intercourse, and is highly prevalent in regions where HPV vaccine is not widely used, especially in developing countries [1]. Many treatment options are available for cervical cancer, including surgical resection, radiotherapy, chemotherapy, and biologically targeted therapy; however, cervical cancer still has a high mortality rate [2]. Therefore, we aim to establish a strong prognostic index and new treatment goals for improving patient survival. This should be closely monitored and adjusted for the treatment regimen. Recently, RNA methylation has been found to play an important role in cell proliferation, tumour progression, and prognosis [3]; however, its prognostic role in cervical cancer and its role in drug therapy have not been fully elucidated.

There are multiple transcriptional modifications of RNA [4]. The three most common RNA methylation modifications are m6A, m5C and m1A, which usually regulate gene expression at the post-transcriptional level, and the regulatory molecules are mainly methyltransferases, demethylases and methyl-binding proteins [5–7]. A recent study showed that m6A mRNA methylation plays an important role in cancer development, s such as bladder cancer [8] and stomach cancer [9]. A previous study showed the potential biological function of NSUN2, a regulator of m5C, in common gynaecological cancers, where NSUN2 promotes cervical cancer cell migration and invasion by causing m5C methylation of keratin 13 (KRT13) transcripts [10]. Zhao et al. [11] revealed that dysregulation of m1A enzymes in tumour samples from patients with five gastrointestinal (GI) cancers is associated with multiple types of genetic alterations. However, the role of the three common types of RNA methylation modifications in cervical cancer remains unknown. Therefore, it is crucial to explore the mechanisms of the roles of genes regulated by m6A, m5C and m1A methylation modifications in the prognosis and treatment of cervical cancer.

We downloaded the mRNA expression profiles and clinical information of 306 CESC patients and three normal patients from the public database TCGA in our research. The m6A/m5C/m1A variants were downloaded from the RMVar database, the m6A/m5C/m1A regulators were obtained from the literature, and a total of 545 mRNA methylation-related genes were considered for differential analysis. Using one-way ANOVA, univariate Cox regression, and multivariate COX analyses, we obtained a risk score model with 10 moderators to quantify the methylation modification pattern of each CESC patient. Infiltration analysis of immune cells and scoring of immunosuppressive agents to infer responses and targets for immunotherapy. The IC50 values of the potential chemotherapeutic agents were screened to identify potentially effective agents. We developed an accurate survival risk stratification model (CESC) based on m6A/m5C/m1A-related genes in cervical cancer patients using internal and external validation and survival analysis. This study demonstrates the importance of m6A, m5C and m1A methylation gene models for the prognosis of cervical cancer patients and the establishment of new treatment options.

## Materials and methods

### Cervical cancer data source and pre-processing

Gene expression and mutation data were downloaded from TCGA database for 309 patients with CESC using the 4.2.0. R package ‘TCGA biolinks.’ Additionally, gene expression data for 79 samples in GSE39001 were downloaded from GEO Database. The m6A, m5C, and m1A variants were downloaded from the RMVar database (https://rmvar.renlab.org/index.html), and the m6A/m5C/m1A regulators were obtained from the literature, resulting in a total of 545 mRNA methylation-related genes obtained for follow-up analysis. The technology roadmap for this study is presented in Supplementary Table 1.

### Differential analysis and functional enrichment analysis of methylation-related genes

m6A/m5C/m1A methylation-regulated genes and related variants were analyzed for expression differences, and a mutation analysis with thresholds of |log_2_FC|>0.5 and FDR < 0.05 was used to obtain methylation-related differently expressed genes (MEGs). The MEGs were analyzed for Gene Ontology (GO) and Kyoto Encyclopedia of Genes and Genomes (KEGG) enrichment using the ‘cluster profiler’ R package. Gene Set Enrichment (GSEA) analysis of ggplot2 [version 3.3.3].

### Construction of m6A/m1A/m5C prognostic model

We performed univariate COX analysis on MEGs, screened 21 differentially expressed genes, and generated forest plots. Then, the expression of differentially expressed genes and clinical information were integrated to obtain a methylation model containing the 10 most prognostic genes associated with overall survival (OS) by multifactorial COX analysis. The model risk score was calculated based on the risk coefficients of the 10 genes, and the high-risk and low-risk groups were divided according to the median risk score. Heat maps of the model genes were drawn using the ‘pheatmap’ package.

### Internal and external validation of the model

Internal validation: Validation and calibration curves were constructed using the Nomgram model. Next, prognostic line plots were generated to predict the 1-, 3-, and 5-year OS of patients with CESC in TCGA. The calibration chart is validated using the TCGA internal and displayed graphically. External validation: The risk score was calculated using the sum of the expression of the 10 model genes in GSE39001, and the multiplication of the risk factors yielded the prognostic model of GSE39001.

### Correlation analysis of clinicopathological features

The ggpubr package [version 0.4.0] was used to assess survival time in high- and low-risk groups at different clinicopathological stages.

### Expression and mutation of m6A/m1A/m5C model genes

Analysis of differential expression of model genes in high- and low-risk groups was carried out. Furthermore, we performed analyses of model gene expression in cervical cancer and paracancer, and mutation status and survival analysis of different groups.

### Immunofunctional analysis of m6A/m5C/m1A-related gene models

Using CIBERSORT, we analyzed 22 immune cell infiltrates in the models. ssGSEA [version 1.44.3] was used to analyze the expression of immune functions. Analysis of the expression of seven immunodetection site inhibitors and correlation analysis of 10 model genes with immune cells was performed.

### Predicting patient response to immunotherapy and screening potential therapeutic agents

We downloaded the immunophenotypic score (IPS) for cervical cancer from the Cancer Immunome Atlas (https://tcia.at/). We compared the differential expression of anti-PD-L1, anti-PD, and anti-CTLA-4 in high- and low-risk subgroups. This was used to infer the treatment response to immune checkpoint inhibitors (ICIs). We used the pRRophetic_0.5 package to predict potential therapeutic agents.

### Validation of protein expression levels of key prognostic genes by immunohistochemistry and mRNA levels by RT-qPCR

As SLC2A1 and CA2 are highly expressed in cervical cancer tissues and are high-risk factors in the model, and CUX1 is under-expressed in cervical tissues and is a high-risk factor in the model, these genes are closely related to the prognosis of cervical cancer. We selected three key prognostic genes and performed immunohistochemistry and RT-qPCR validation of the SLC2A1, CUX1, and CA2 genes. Samples of 30 cervical cancer tissues and their parametrial tissues were collected from January 2022 to August 2022 at the First Affiliated Hospital of Bengbu Medical College, where the patients were hospitalized for surgery. We obtained the patient’s informed consent. A portion of the retained tissue was used to extract nucleic acids using the Biosharp BS259A Total RNA Extraction Kit. The mRNA expression of SLC2A1, CUX1, and CA2 was detected using the RT-qPCR method (PCR primers are in Supplementary Table 2). Another portion of the tissue was made into paraffin sections and stained for IHC. Antibodies used were as follows: Anti-SLC2A1 (21829-1-AP, proteintech), Anti-CUX1 (11733-1-AP, proteintech), and Anti-CA2 (16961-1-AP, proteintech). The secondary antibodies used were goat anti-rabbit IgG and HRP. The expression intensity ranged from strong to weak, pale yellow (+), yellow (++), and brown (++++). All IHC results were evaluated by two independent pathologists. Ethical approval for this study was granted by the Clinical Medical Research Ethics Committee of the First Affiliated Hospital of Bengbu Medical College (Bengbu, China). Lundin Mobile Science Grant [2022] No. 343.

### Statistical analysis

Statistical analyses were performed using R software (4.2.0), and continuous variables in both groups were analyzed by *t*-test or Mann–Whitney U test. COX, LASSO, and K-M survival analyses were used to identify m6A/m1A/m5C regulatory genes associated with the prognosis of CESC patients. *p* < .05 was considered a statistically significant difference.

## Results

### Differential expression and mutation of m1A/m5C/m6A regulatory genes in cervical cancer

**Figure 1.**
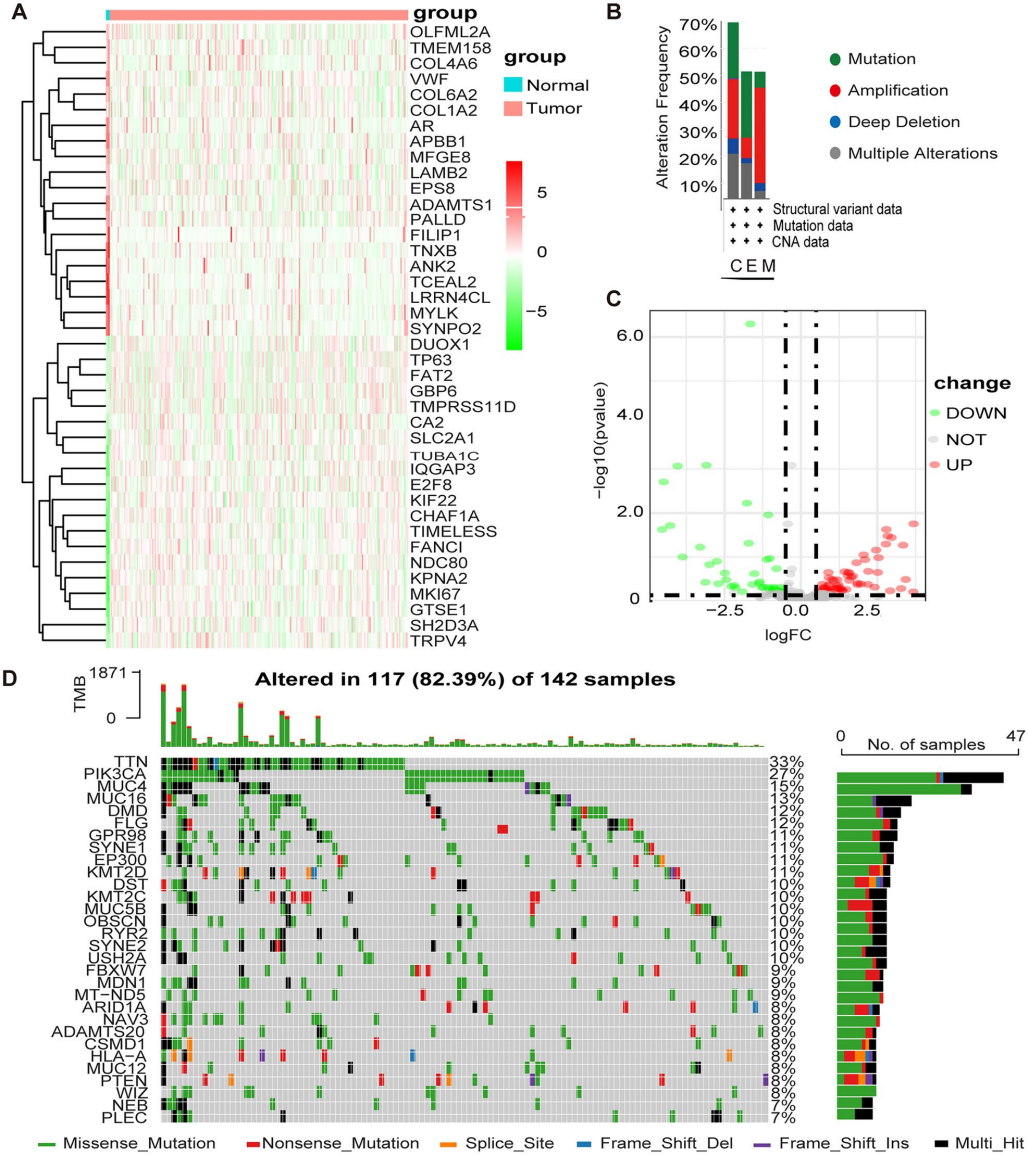
Differential expression and mutation of m1A + m5C + m6A regulatory genes in cervical cancer. (A) Heatmap of MEGs. Green represents down-regulation and red represents up-regulation of the gene. (B) Up-regulated gene mutations Mutation sites for mutations in the mutation frequency. (C) Volcano map of MEGs. Green dots represent 44 down-regulated genes; red dots represent 62 up-regulated genes. (D) 117 of the 142 CESC patients experienced genetic alterations of m6A, m5C, m1A regulators, with a frequency of 82.39%, mostly including amplification, missense mutations, and deep deletions. The number on the right indicated the mutation frequency in each regulator.

The clinical data and expression profiles of 306 patients with CESC and three normal patients were obtained from TCGA, the Baseline Information Sheet in Supplementary Table 3. The differential analysis yielded 106 methylation-related differential genes (MEDS), including 44 differentially expressed genes and 62 upregulated genes. Figure 1(A) shows the heat map of the top 20 up- and down-regulated MEDS, and Figure 1(C) shows the volcano map of the top 20 up- and down-regulated MEDS. The genes with higher mutation frequency in MEGs, in descending order, were TTN, PIK3CA, MUC4, MUC16, DMD, and FLG (Figure 1(D)). The variations included amplifications, deletions, mutations, and complex alterations. The largest proportion of mutation frequency was found in the up-regulated gene set of endocervical adenocarcinoma (Figure 1(B)).

### Construction of the m1A + m5C + m6A methylation model

**Figure 2.**
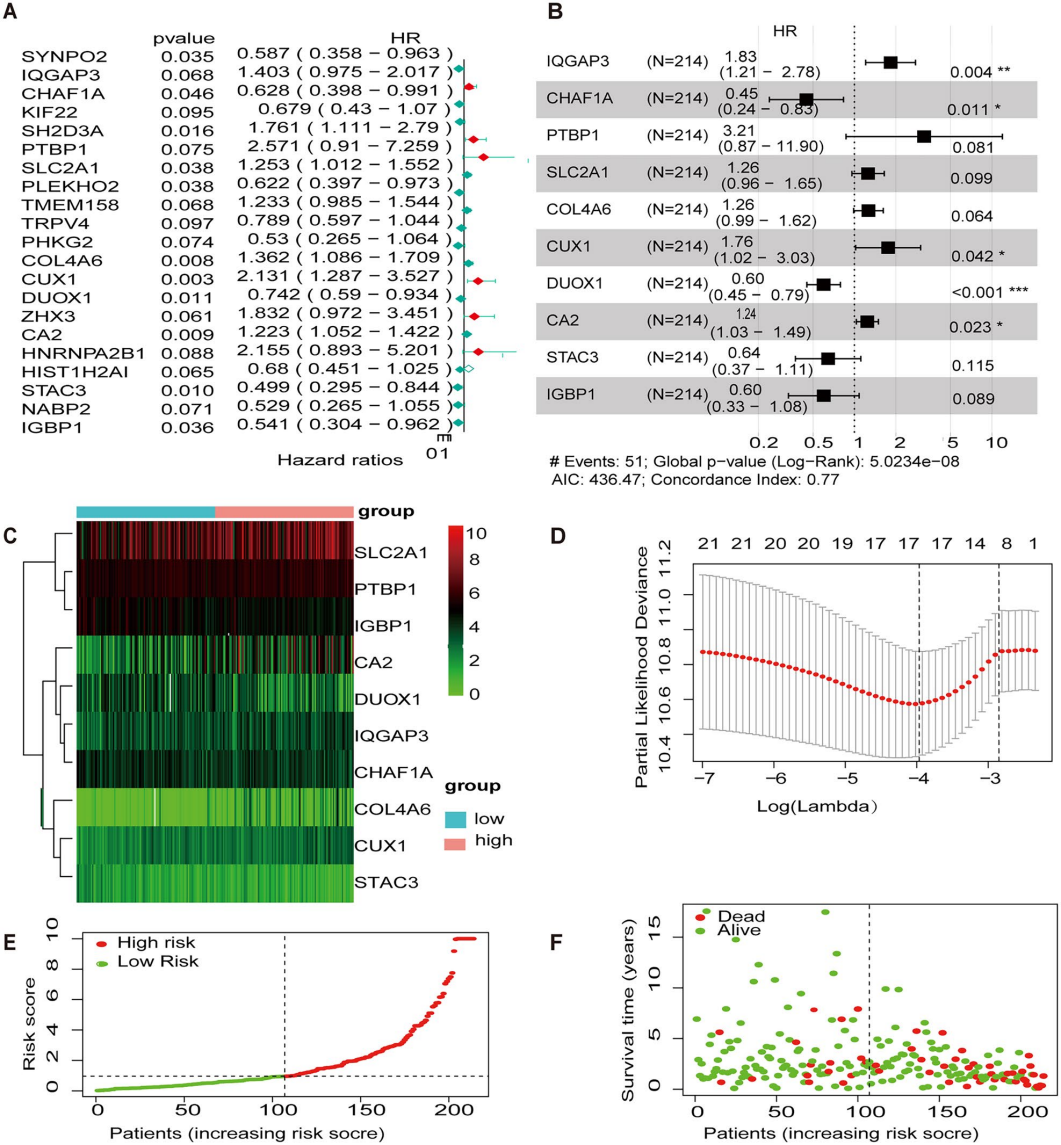
Development of prognostic features of the TCGA-CESC methylation model. (A) Results of univariate cox analysis of OS. (B) Results of multifactorial cox analysis of OS. (C) LASSO coefficient profiles of 10 methylation-associated genes. (D) Heat map of 10 differential gene expressions between high- and low-risk scoring groups. (E) Risk score scatter plot. Red dots indicate dead patients and green dots indicate living patients. (F) Risk score curve graph. Green curves indicate low-risk group and red curves indicate high-risk group.

The expression profiles of 106 MEGs were combined with clinical survival information for univariate cox regression analysis. Twenty-one MEGs were identified as associated with OS in patients with CESC (*p* < .1) (Figure 2(A)). LASSO analysis was performed to select the best prognostic genes associated with OS (Figure 2(B)). The correlation coefficients and risk score of the prediction model were calculated, and 10 methylation-related regulators were finally identified with HR > 1 for high-risk molecules, including IQGAP3, PTBP1, STAC3, CUX1, SLC2A1, and CA2, and HR < 1 for low-risk molecules, including IGBP1, DUOX1, CHAF1A, and STAC3 (Figure 2(C)). Survival status and risk profile charts indicate a worse prognosis in the high-risk group (Figure 2(E), F). The results of the univariate and multifactor COX regression analyses are presented in (Table 1).

**Table 1.** Univariate and multivariate COX analysis.

Characteristics	Total (*N*)	Univariate analysis		Multivariate analysis	
		Hazard ratio (95% CI)	*p* Value	Hazard ratio (95% CI)	*p* Value
T stage	241				
T1	138	Reference			
T2	72	1.109 (0.477–2.578)	0.810	0.000 (0.000-Inf)	0.999
T4	10	10.411 (4.231–25.618)	<0.001		
T3	21	3.251 (1.274–8.292)	0.014	2.470 (0.268–22.747)	0.425
N stage	193				
N0	133	Reference			
N1	60	3.544 (1.572–7.987)	0.002	3.319 (0.967–11.398)	0.057
M stage	125				
M0	114	Reference			
M1	11	4.290 (1.401–13.137)	0.011	0.025 (0.000-Inf)	1.000
Clinical stage	295				
Stage I	158	Reference			
Stage II	69	0.895 (0.417–1.921)	0.776	0.000 (0.000-Inf)	0.999
Stage III	46	1.570 (0.732–3.366)	0.247	0.000 (0.000-Inf)	0.999
Stage IV	22	5.109 (2.559–10.199)	<0.001		
Age	302				
< =50	186	Reference			
>50	116	1.295 (0.761–2.204)	0.340		
Histological type	302				
Adenosquamous	52	Reference			
Squamous cell carcinoma	250	0.983 (0.481–2.011)	0.963		

### Internal validation of methylation models in the TCGA-CESC

**Figure 3.**
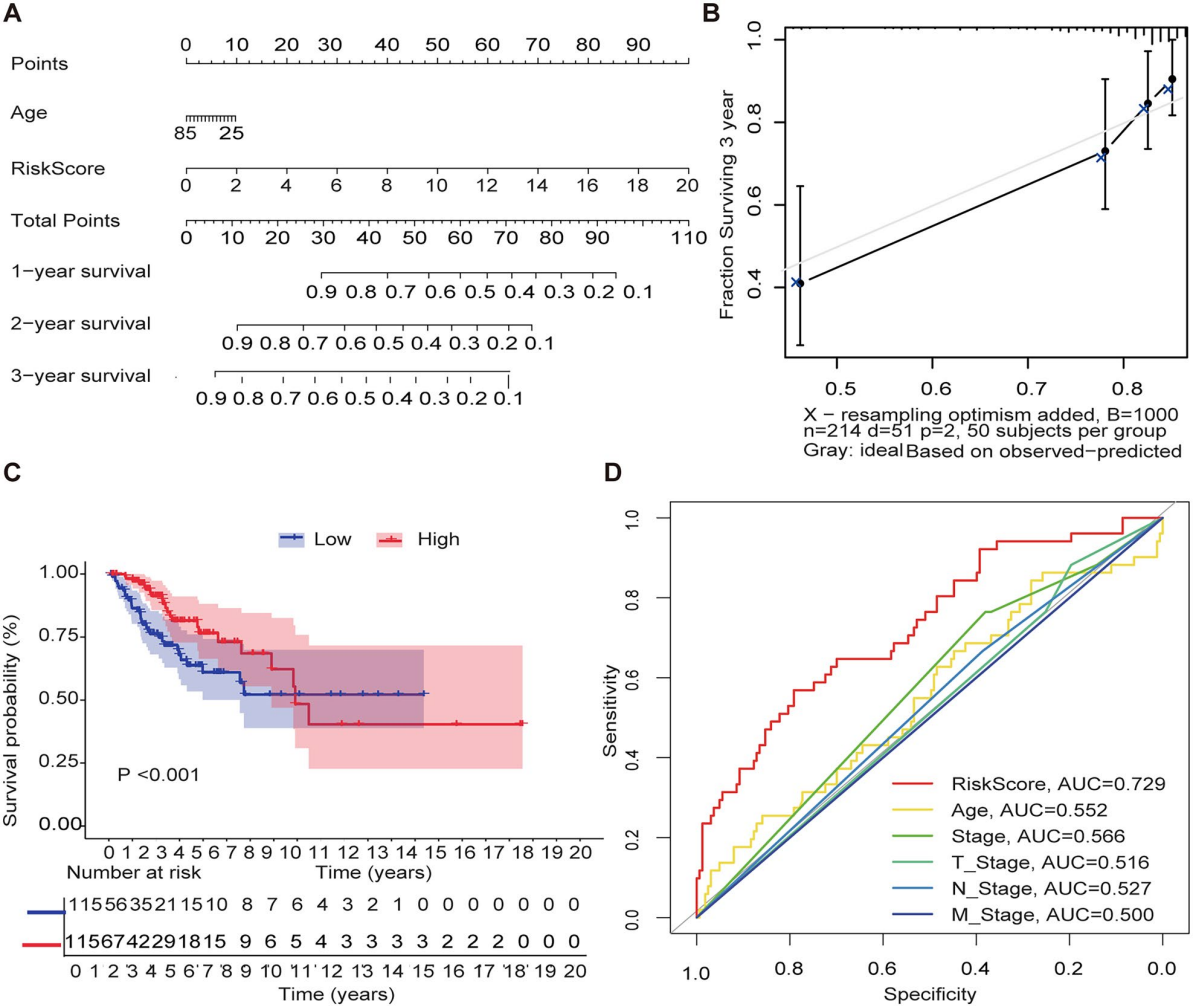
Internal validation of methylation models by TCGA-CESC. (A) Establishing a column line graph based on prognostic features to predict OS in cervical cancer in the TCGA-CESC. (B) Calibration curve for 3-year column line graph prediction. (C) Kaplan–Meier survival curves. Survival time was shorter in the high-risk group in the TCGA-CESC. (D) ROC Curve in the TCGA-CESC.

We used the Nomgram model constructed using age and risk scores to assess survival time at 1 year/3 years/5 years (Figure 3(A)). In addition, corrected with a 3-year calibration curve, the 3-year OS and the probabilities predicted by the column line plot are consistent with the actual proportions (Figure 3(B)). The results of the ROC curve analysis indicate that the risk score has better predictive power (AUC = 0.729) than other relevant clinical parameters (Figure 3(D)).

### External validation of methylation model in GSE39001

**Figure 4.**
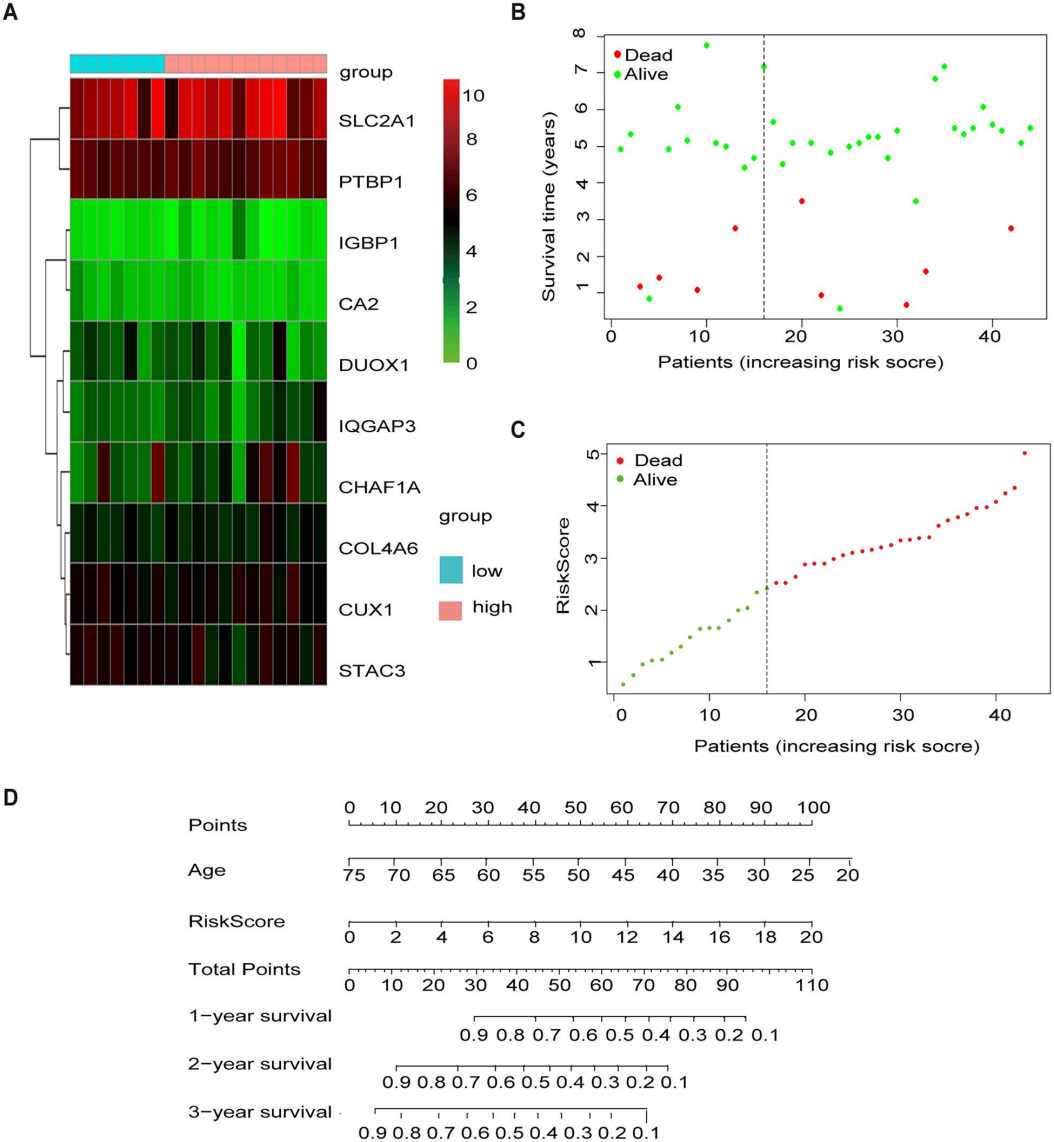
External validation of methylation models by GSE3900. (A) Heat map of 10 differential gene expressions between high- and low-risk scoring groups in GSE39001. (B) Risk score scatter plot. Red dots indicate dead patients and green dots indicate living patients in GSE39001. (C) Risk score curve graph in GSE39001. Green curves indicate low-risk group and red curves indicate the high-risk group. Alive. (D) Establishing a column line graph based on prognostic features to predict OS in cervical cancer in GSE39001.

To validate the methylation model in the exogenous database in terms of predictive performance, the GSE39001 risk score was calculated using the same formula as for patients in TCGA, and we mapped the heat maps for 10 genes in the GEO cohort in the high- and low-risk groups (Figure 4(A)). Similarly, the risk profile and survival status plots in the GEO cohort highlighted the poorer overall survival in the high-risk group (Figure 4(B,C)), which is consistent with TCGA model results. We also used the Nomgram model constructed using age and risk scores to assess survival time at 1 year/3 years/5 years (Figure 4(D)).

### Functional enrichment analysis of MEGs

**Figure 5.**
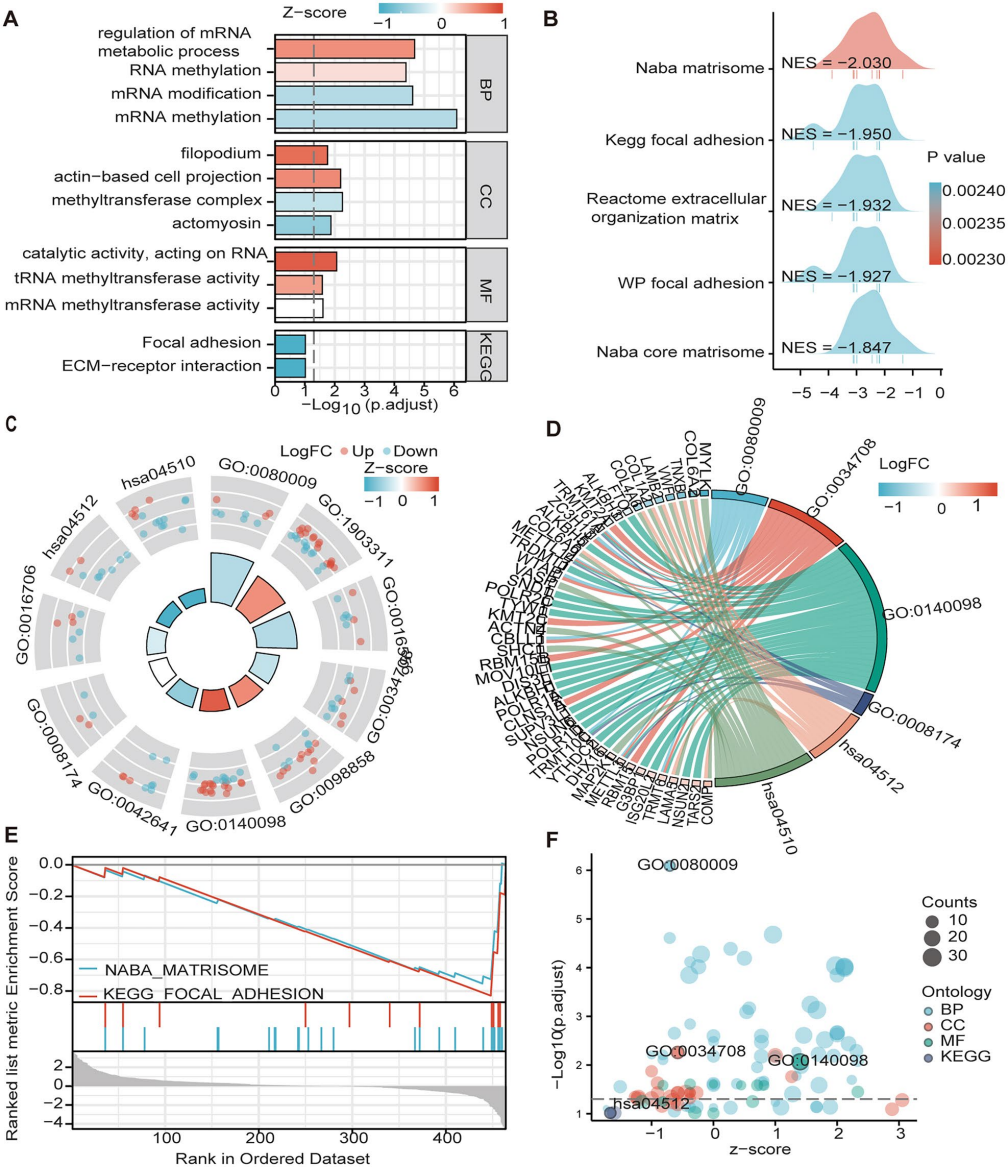
The GO/KEGG/GSEA enrichment analysis on the MEGs (A\C\D\F) Histograms\circles\bubbles\chord plots for en GO/KEGG enrichment analysis. (B) Top 5 GSEA enrichment analyses on the MEGs (E)GSEA enriched gene set in the C2 set, KEGG gene set and NABA gene set.

GO analysis showed that the enrichment of MEGs was mainly related to mRNA methylation. For example, BP was mainly enriched in mRNA methylation, regulation of mRNA metabolic process; macromolecule methylation; mRNA metabolic process; macromolecule methylation and mRNA modification. CC is enriched in methyltransferase complex, actin-based cell projection and filopodium. MF enriched in mRNA/tRNA/RNA methyltransferase activity. Similarly, KEGG enrichment analysis revealed enrichment in the ECM-receptor interaction and focal adhesion pathways (Figure 5(A/C/D/F) and Table 2). The GSEA step explored the potential molecular mechanisms of MEGs in TCGA cohort. MEGS analysis in GSEA revealed that differential genes were mainly enriched in cell cycle, signal transduction, and cancer pathways, including reactome cell cycle, Kegg focal adhesion, reactome extracellular matrix organization, reactome post-translational protein modification, and reactome transcriptional regulation by tp53 and the wp pi3kakt signaling pathway (Figure 5(B,E) and Table 3).

**Table 2.** GO and KEGG analysis.

Ontology	ID	Description	Gene Ratio	Bg Ratio	*p* Value	*p* adjust	*q* Value
BP	GO:0080009	mRNA methylation	8/433	14/18670	2.09e-10	8.15e-07	7.72e-07
BP	GO:1903311	regulation of mRNA metabolic process	27/433	324/18670	1.07e-08	2.10e-05	1.99e-05
BP	GO:0001510	RNA methylation	13/433	81/18670	4.21e-08	4.11e-05	3.90e-05
BP	GO:0043414	macromolecule methylation	25/433	309/18670	6.77e-08	5.29e-05	5.01e-05
CC	GO:0034708	methyltransferase complex	12/450	113/19717	1.06e-05	0.006	0.005
CC	GO:0098858	actin-based cell projection	16/450	208/19717	2.46e-05	0.006	0.005
CC	GO:0042641	actomyosin	9/450	79/19717	7.76e-05	0.013	0.011
CC	GO:0030175	filopodium	10/450	104/19717	1.34e-04	0.017	0.015
CC	GO:0030018	Z disc	11/450	132/19717	2.25e-04	0.023	0.020
MF	GO:0140098	catalytic activity, acting on RNA	25/438	386/17697	1.26e-05	0.009	0.008
MF	GO:0008174	mRNA methyltransferase activity	4/438	11/17697	1.06e-04	0.025	0.023
MF	GO:0008175	tRNA methyltransferase activity	6/438	34/17697	1.66e-04	0.026	0.024
MF	GO:0008173	RNA methyltransferase activity	8/438	66/17697	2.16e-04	0.026	0.024
KEGG	hsa04512	ECM-receptor interaction	9/210	88/8076	4.41e-04	0.096	0.090
KEGG	hsa04510	Focal adhesion	14/210	201/8076	7.31e-04	0.096	0.090

**Table 3.** GSEA analysis.

ID	Set Size	Enrichment score	NES	*p* Value	*p*. adjust	*q* Value
reactome_cell_cycle_checkpoints	237	0.6667500	3.043798	1e-10	7.58e-09	6.02e-09
reactome_mitotic_g1_phase_and_g1_s_transition	142	0.7009502	3.002095	1e-10	7.58e-09	6.02e-09
reactome_DNA_replication	137	0.6958208	2.956086	1e-10	7.58e-09	6.02e-09
reactome_cell_cycle_mitotic	458	0.6073098	2.947152	1e-10	7.58e-09	6.02e-09
cell_cycle__G2_M_checkpoints	134	0.6866194	2.901270	1e-10	7.58e-09	6.02e-09
reactome_synthesis_of_DNA	110	0.7113475	2.900659	1e-10	7.58e-09	6.02e-09
reactome_mitotic_metaphasee_and_anaphase	201	0.6502340	2.887159	1e-10	7.58e-09	6.02e-09
WP_retinoblastoma_gene_in_cancer	84	0.7301521	2.814346	1e-10	7.58e-09	6.02e-09
reactome_s_phase	145	0.6553133	2.798761	1e-10	7.58e-09	6.02e-09
reactome_mitotic_spindle_checkpoint	92	0.7015493	2.772420	1e-10	7.58e-09	6.02e-09

### Gene expression and clinical characteristics based on methylation model

**Figure 6.**
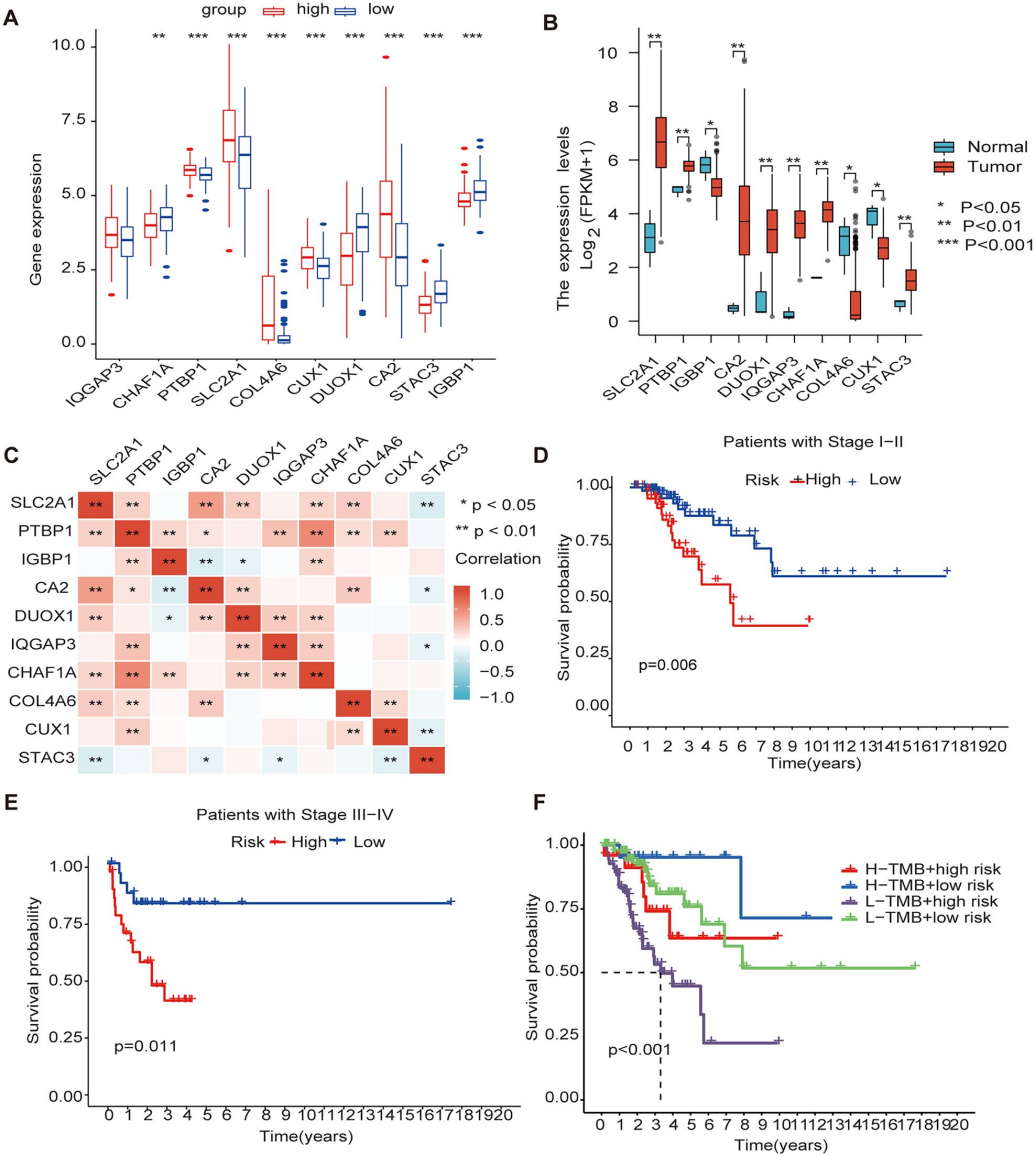
Gene expression and clinical characteristics based on m6A/m5C/m1A methylation model (A) The model gene expression in high and low-risk groups. (B) All ten regulators were significantly expressed in patients with CESC compared with the normal counterparts, of which seven regulators (SLC2A1, PTBP1, CA2, DUOX1, IQGAP3, CHAF1A, STAC3) were significantly up-regulated, and three regulators (IGBP1, CUX1, COL4A6) were significantly down-regulated. (C) Analysis of Pearson correlation of 10 model genes in CESC. (D) Kaplan–Meier curves for Clinical stage I-II. (E) Kaplan–Meier curves for Clinical stage I-II III-IV. (F) K-M analysis of each high and low mutation group in the model high and low-risk groups.

The methylation model revealed that the genes highly expressed in the high-risk group were SLC2A1, PTBP1, COL4A6, CUX1, and CA2; the genes highly expressed in the low-risk group were CHAF1A, DUOX1, STAC3, and IGBP1 (Figure 6(A)); The genes highly expressed in the cancer tissue were PTBP1, CA2, DUOX1, IQGAP3, CHAF1A, and STAC3; and genes with high expression in the normal adjacent tissue were CUX1, IGBP1, and COL4A6 (Figure 6(B)). Meanwhile, We have made a Pearson correlation analysis for the 10 MEGs genes. Deep red color indicates a strong positive correlation and a deep blue color indicates a strong negative correlation, and the results showed that SLC2A1 was significantly correlated with PTBP1, CA2, DUOX1, CHAF1A and COL4A6, respectively, and PTBP1 was significantly correlated with IGBP1, IQGAP3, CHAF1A, CLO4A6 and CUX1, respectively (Figure 6(C)). The comparison of survival time in different clinical stages revealed that the survival time in stages I-II and III-IV in the high-risk group was lower (Figure 6(D–E)). Mutation analysis of model genes revealed no statistically significant difference in mutation frequency between the high- and low-risk groups However, the low mutation group had a worse prognosis than the high mutation group, and the high-risk low mutation rate group had a shorter survival time and a worse prognosis than the other groups (Figure 6(F)). A prognostic analysis of the model genes is presented in Supplementary Figure 1.

### Immune cell infiltration in the m1A/m5C/m6A prognostic model

**Figure 7.**
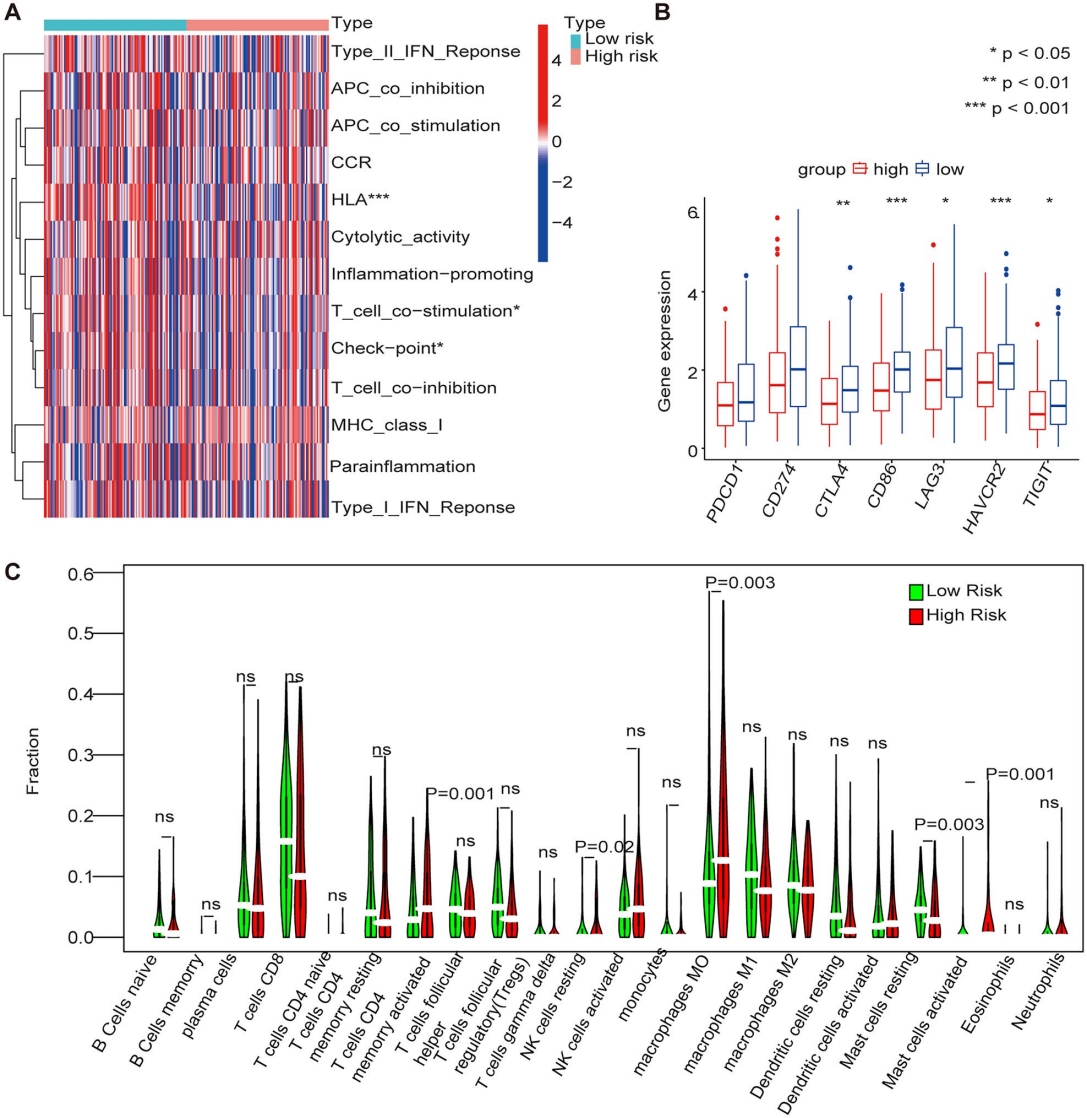
Immune cell infiltration in the m1A + m5C + m6A prognostic model (A) The heat map shows the comparison of immune-related functions in the high and low-risk groups. (B) The expression of immune checkpoint inhibitors (ICIs) in high and low-risk groups. (C) The violin plot shows the distribution of 22 immune cell infiltrates in the high and low-risk groups of the methylation model.

The immune cell infiltrations in the high-risk group were mase cells activated and mase cells resting, and immune cell infiltrations in the low-risk group were regulatory T cells (Tregs), resting NK cells, macrophages M0 and dendritic cells (Figure 7(C)). The immune functions that were highly expressed in the low-risk group were HLA, T cell co-stimulation, and checkpoints (Figure 7(A)). The expression of immune checkpoint inhibitors (ICPs) in the high- and low-risk groups were analyzed, and the results showed that high expression of ICPs such as cytotoxic T lymphocyte antigen 4 (CTLA4), CD86, lymphocyte activation gene-3 (LAG3), HAVCR2, and TIGIT was observed in the low-risk group (Figure 7(B)).

### Immunosuppressant treatment response and potentially sensitive drugs

**Figure 8.**
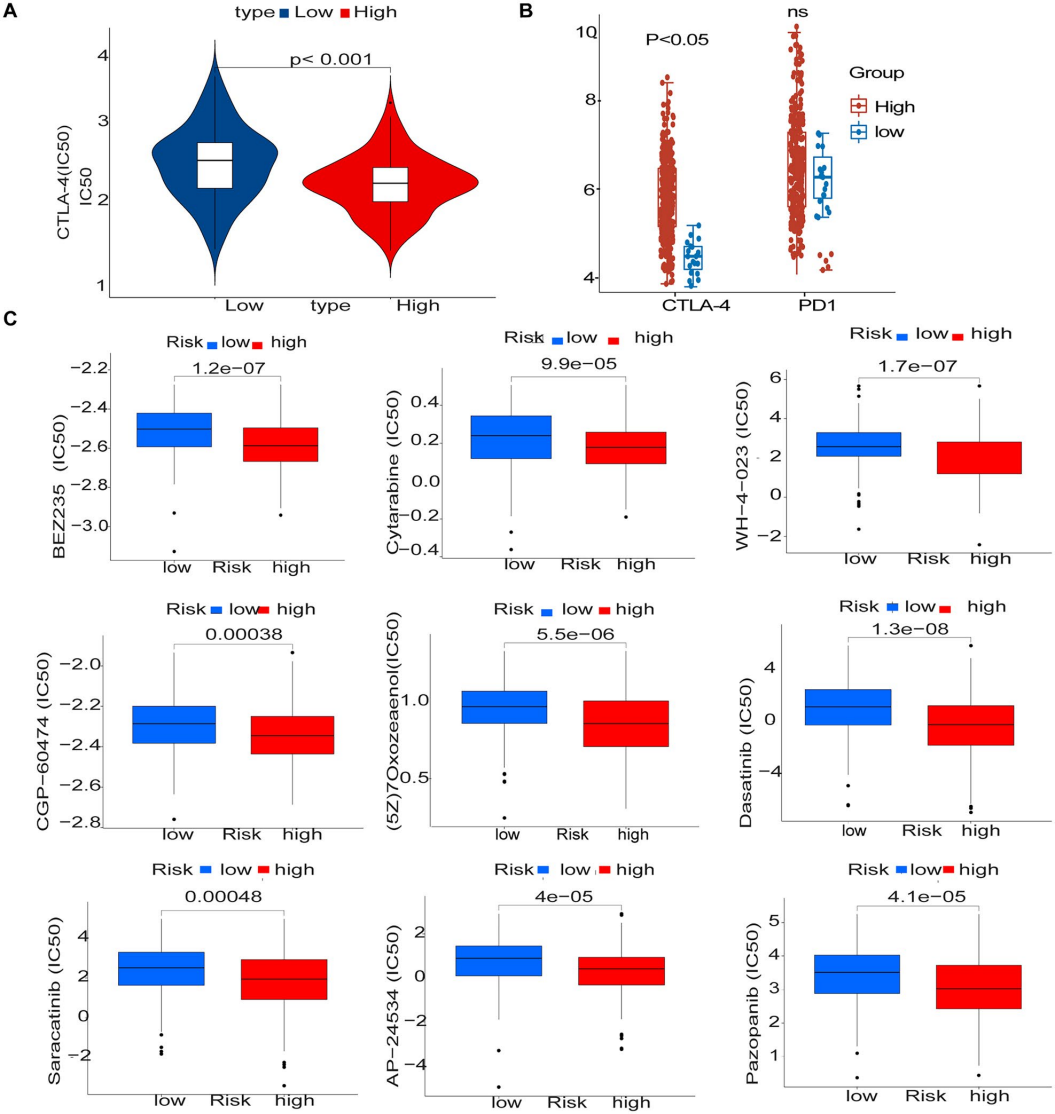
Immunosuppressant treatment response and potentially sensitive drugs (A) IC50 values for anti-CTLA-4 were smaller in the high-risk group, and patients in the high-risk group may be suitable for anti-CTLA-4 therapy (B) The high and low-risk score groups responded to anti-PD-1/PD-L1 and anti-CTLA-4 therapy. (C) Box plot showing differential expression of IC50 for 9 drugs in high and low-risk groups.

We used two immunophenotypic scores (IPS) subtypes (IPS-PD-1/PD-L1/PD-L2_pos and IPS-CTLA-4_pos) as proxies for response to anti-PD-1/PD-L1 and anti-CTLA-4 treatment in patients with CESC. The results suggest that the relative probability of response to anti-CTLA-4 treatment was higher in the high-risk group according to the methylation prediction model (*p* < .05) (Figure 8(B)). The IC50 values for anti-CTLA-4 were lower in the high-risk group, and patients in the high-risk group may be suitable for anti-CTLA-4 therapy (Figure 8(A)). Drugs were screened for potential treatment using the pRRophetic_0.5 package, and the horizontal coordinates of the box plot indicate the risk, with blue indicating low risk, red indicating high risk, and the vertical coordinates indicating the IC50 values of the drugs. We screened nine drugs ((5Z)-7-Oxozeaenol, AP-24534, BEZ235, CGP-60474 cytarabine, dasatinib, Pazopanib, saracatinib, and WH-4-023) and they had lower IC50 values in the high-risk group (*p* < .05), confirming their higher efficacy in the high-risk (Figure 8(C)). Correlation analysis showed that the nine drugs were positively correlated with the genes in the high-risk group (Supplementary Figure 2).

**Figure 9.**
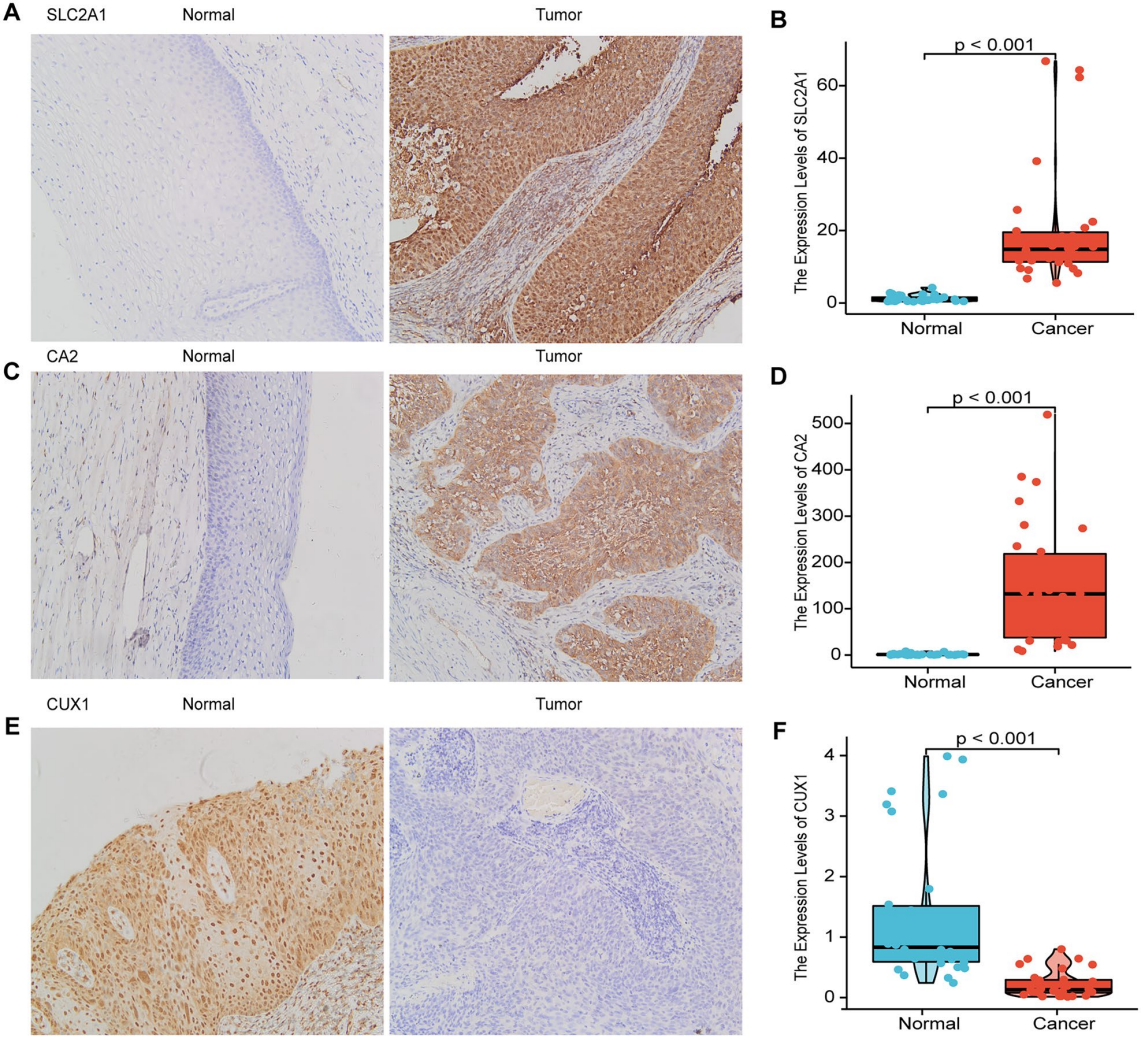
Immunohistochemical validation and RT-qPCR validation of key prognostic genes. Immunohistochemical results showed that SLC2A1 and CA2 were highly expressed in cervical cancer tissues, and CUX1 was lowly expressed in cervical cancer tissues (A, C, E), RT-qPCR results showed that SLC2A1 and CA2 were highly expressed in cervical cancer tissues, and CUX1 was lowly expressed in cervical cancer (B, D, F).

### Immunohistochemical validation and RT-qPCR validation of key prognostic genes

Therefore, we used immunohistochemistry and RT-qPCR analyses of clinical samples to verify the expression of SLC2A1, CUX1, and CA2. SLC2A1 and CA2 were highly expressed, and CUX1 was weakly expressed, in cervical cancer tissues, as determined by immunohistochemistry (Figure 9(A,C,E)) and RT-qPCR (Figure 9(B,D,F)). SLC2A1 positive sites were positive in the cell membrane, cytoplasm and nucleus, CA1 positive sites were predominantly in the cell pulp and partly in the nucleus, and CUX1 was predominantly positive in the nucleus.

## Discussion

The prognosis of malignant tumors is associated with changes in the expression of multiple genes [12–15]. Liu et al. [12] found that SEMA3C strongly correlates with a shorter survival time in cervical cancer. Griesmannd et al. [13] found that the cut-like homology cassette 1 (CUX1) enhances pancreatic cancer proliferation by increasing the activation of MEK-ERK signaling upstream of the ADAM17 protein. Aly et al. [14] found that somatic mutations CUX1 gene can be found in myeloid neoplasms (MNs), Especially in myelodysplastic syndromes (MDSs), which also leads to DNA repair dysfunction, CUX1MT/DEL is also associated with poorer survival rates. Zhang et al. [15] found that CA2 inhibits tumor metastasis in HCC that is dependent on the α1 subunit of ATP1A1 in inpatients with hepatocellular carcinoma.

Some studies have found that modifications in RNA methylation are associated with the progression of cervical cancer. Xie et al. [16] found that high piRNA-14633 expression in cervical cancer promotes CC cell proliferation, migration, and invasion. Furthermore, Lin et al. [17] found that m6A modifications of CENPK mRNA are regulated by ZC3H13. For example, elevated CENPK expression in cervical cancer was associated with cancer recurrence and independently predicted poor patient prognosis. Zou et al. [18] Found that SLC2A1 has abundant methylation sites and was associated with immunosuppression in colon cancer. High expression of SLC2A1 in high-grade CIN with high-risk human papillomavirus (HR-HPV) infection indicates a high risk of cervical cancer [19]. This study also confirms that SLC2A1 and CA2 are highly expressed, and that CUX1 is weakly expressed, in cervical cancer tissues. They are all highly expressed in the model and are high-risk genes that correlate with poor prognosis, indicating that the m6A/m1A/m5C regulatory genes play an important role in cervical cancer progression. In this study, we investigate the role of m6A, m1A, and m5C regulatory genes in cervical cancer progression, and obtained 21 MEGs associated with OS in patients with CESC by univariate Cox regression analysis of differential genes (*p* < .1). We performed LASSO analysis to select the best prognostic genes associated with OS and calculated the correlation coefficients of prediction models with risk Score. The final 10 methylation-related regulators with HR >1 were identified as high-risk molecules, including IQGAP3, PTBP1, COL4A6, CUX1, SLC2A1, and CA2, and those with HR < 1 were considered low-risk molecules, including IGBP1, DUOX1, CHAF1A, and STAC3. The ROC curve shows that the risk score of the model exhibits good predictive performance (AUC = 0.729). In the model, the high-risk group demonstrated poor overall survival. Mutation analysis of the model genes revealed that the high-risk low mutation rate group had a lower survival time and worse prognosis than the other groups. Through our study, we found that m6A, m1A and m5C regulatory genes play an important biological role in the progression of CESC. These markers might be used to detect the prognosis and survival time of CESC patients.

KEGG and GSEA analyses were mainly enriched in the ECM-receptor interaction, focal adhesion, and wp pi3kakt signalling pathway, and they were closely associated with cancer progression [20–22]. Zhang et al. [20] found that metformin significantly inhibits the PI3K/AKT signaling pathway in CaSki, C33A, and HeLa cells, and induces apoptosis and cell cycle arrest in human cervical cancer cell lines (CaSki and HeLa). Hao et al. [21] found that miR-7 inhibits the metastasis and invasion of cervical cancer by targeting focal adhesion kinase (FAK). Ma et al. [22] found that downregulation of COL4A6 promotes prostate cancer progression and invasion and that COL4A6 and its promoter methylation status are important markers of prostate cancer prognosis. In this study, COL4A6 was found to be under-expressed in cervical cancer tissues and is a high-risk prognostic marker. GSEA reveal that COL4A6 may regulate ECM-receptor interaction, focal adhesion, and the wp pi3kakt signaling pathway. The m6A/m1A/m5C regulatory genes in this study are shown to be involved in various functions, previous studies have shown that m6A/m1A/m5C-regulated genes are strongly associated with CESC prognosis. However, the mechanism of the role of m6A/m1A/m5C regulatory genes in the progression of cervical cancer remains to be confirmed by further studies.

Immunotherapy is an important treatment modality for cervical cancer, and Heeren et al. [23] found that the combination of TILs and anti-PD1 significantly improves the prognosis of metastatic cervical cancer. However, only a small proportion of patients with squamous cell carcinoma (SCC) of the uterine cervix derive clinical benefits from ICB therapy [24]. A study by Ari found that 30% of cervical malignancies are positive for cytotoxic T-lymphocyte antigen 4 (CTLA4) [25]. The current study suggests a close correlation between immunotherapy and the immune microenvironment [26]. We found that the risk model constructed by m6A/m1A/m5C regulatory genes was associated with multiple immune cell infiltrations in CESC. The immune cell infiltrations in the high-risk group in the model were resting and activated Mase cells, and in the low-risk group regulatory T cells (Tregs), resting NK cells, macrophages M0 and dendritic cells were prevalent in the correlation and difference analyses. A total of six immune cells was found to be closely related to the model genes. The immune functions that were highly expressed in the low-risk group were HLA, T cell co-stimulation, and checkpoint. ICPs, includingCTLA4, CD86, LAG3, HAVCR2, and TIGIT, were also observed in the low-risk group. However, the relative probability of response to anti-CTLA-4 treatment was higher in the high-risk group in the methylation prediction model. The IC50 values for anti-CTLA-4 were smaller in the high-risk group. Therefore, it is presumed that patients in the high-risk group may be suitable for anti-CTLA-4 therapy.

There are few phase III clinical trials on the use of immunotherapy for cervical cancer [27,28], and preclinical studies have shown that the combination of anti-vascular inhibitors with immune checkpoint inhibitors exerts stronger anti-tumor effects and possesses good clinical potential [29,30]. This study also screened for the potential anti-angiogenic drug pazopanib, which targets the vascular endothelial growth factor receptor (VEGFR) and inhibits neoangiogenesis of the blood supply to the tumor. A phase II study demonstrated the activity of anti-angiogenic drugs in advanced and recurrent cervical cancer, and pazopanib may prolong Progression-Free-Survival (PFS) with fewer toxic effects [31]. Other potential therapeutic agents include 5Z)-7-Oxozeaenol, AP-24534, BEZ235, CGP-60474, cytarabine, dasatinib, saracatinib, and WH-4-023, which has lower IC50s in the high-risk group (*p* < .05), suggesting high-risk cancer have higher efficacy. Correlation analysis showed that the nine drugs were positively correlated with genes in the high-risk group. These genes may provide a basis for targeted therapy for cervical cancer.

## Conclusion

In this study, we identified prognostic models for CESC-related m6A, m5C, and m1A regulatory genes. We validated the mRNA and protein expression levels of SLC2A1, CUX1, and CA2 in clinical samples. Modeled high-risk genes included IQGAP3, PTBP1, STAC3, CUX1, SLC2A1, and CA2, and low-risk genes included IGBP1, DUOX1, CHAF1A, and STAC3. The high-risk group exhibited lower survival times and was associated with immune microcellular infiltration, immune function, and immune checkpoint inhibitors. This study predicts that anti-CTLA-4 therapy may serve as an immunotherapeutic agent for managing cervical cancer and that the expression of CUX1, SLC2A1, and CA2 genes validated by clinical samples may serve as important targets for treatment modalities in cervical cancer. Nine potential target drugs were screened, among which the anti-vascular drug pazopanib may be of significant value. Future studies will allow us to validate the effects of these drugs through further clinical drug trials.

## Supplementary Material

Supplemental Material

Supplemental Material

Supplemental Material

Supplemental Material

Supplemental Material

## Data Availability

All authors confirm that all supporting data, figures, grants, and ethics for this study can be found in the article.
