# Supplementary figures and images for: RNA methylation-related genes of m6A, m5C, and m1A predict prognosis and immunotherapy response in cervical cancer
Source: Ann Med. 2023 Apr 12;55(1):2190618. doi: 10.1080/07853890.2023.2190618 (PMC10101678; doi:10.1080/07853890.2023.2190618)

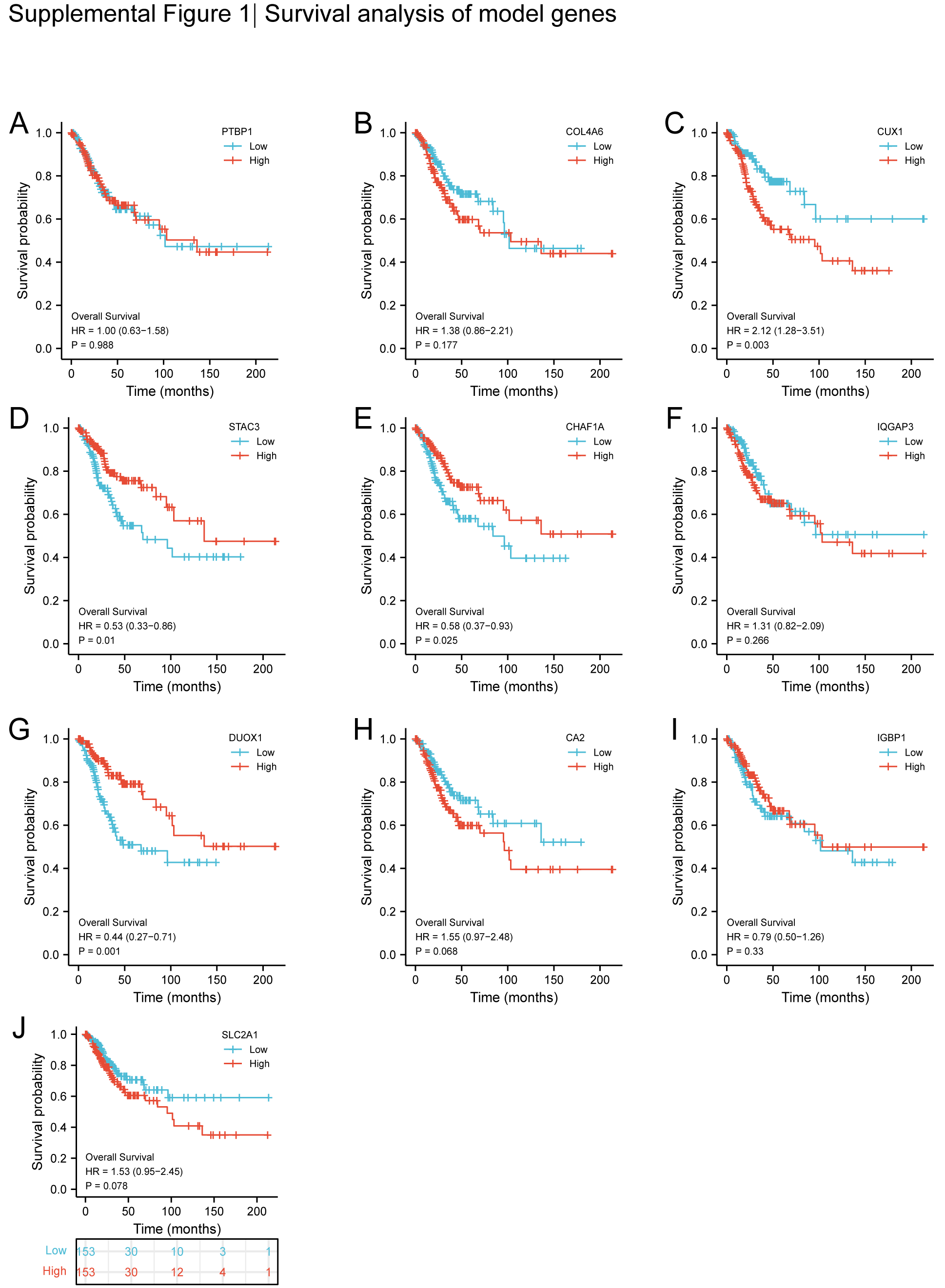

Supplement: Supplemental Material [file IANN_A_2190618_SM7248.tif]

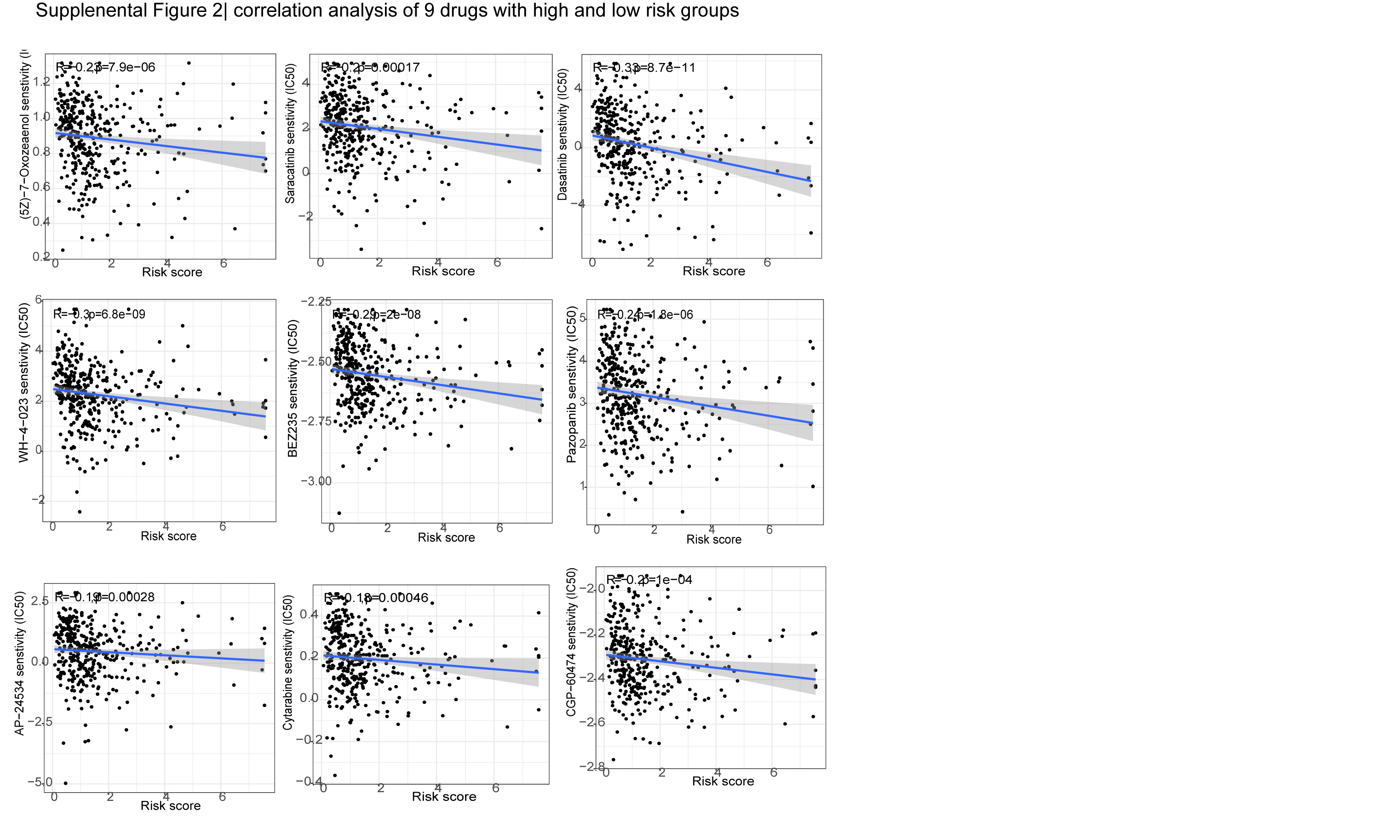

Supplement: Supplemental Material [file IANN_A_2190618_SM7233.tif]
