# Supplementary material for: RNA methylation-related genes of m6A, m5C, and m1A predict prognosis and immunotherapy response in cervical cancer
Source: Ann Med. 2023 Apr 12;55(1):2190618. doi: 10.1080/07853890.2023.2190618 (PMC10101678; doi:10.1080/07853890.2023.2190618)
Supplement: Supplemental Material [file IANN_A_2190618_SM7208.docx]

Supplementary Table3: Baseline Information Table of TCGA-CESC

| Characteristic | levels | TCGA |
| --- | --- | --- |
| n |  | 232 |
| vital_status, n (%) | Alive | 176 (75.9%) |
|  | Dead | 56 (24.1%) |
| N_Stage, n (%) | N0 | 118 (50.9%) |
|  | N1 | 52 (22.4%) |
|  | NX | 62 (26.7%) |
| M_Stage, n (%) | M0 | 101 (43.5%) |
|  | M1 | 11 (4.7%) |
|  | MX | 120 (51.7%) |
| status, n (%) | 0 | 176 (75.9%) |
|  | 1 | 56 (24.1%) |
| age, median (IQR) |  | 46.24 (38.921, 55.473) |
| time, median (IQR) |  | 2.14 (1.298, 3.768) |
