# Supplementary material for: RNA methylation-related genes of m6A, m5C, and m1A predict prognosis and immunotherapy response in cervical cancer
Source: Ann Med. 2023 Apr 12;55(1):2190618. doi: 10.1080/07853890.2023.2190618 (PMC10101678; doi:10.1080/07853890.2023.2190618)
Supplement: Supplemental Material [file IANN_A_2190618_SM7203.docx]

Supplementary Table 2 PCR primers

ID Primer name Sequence(5' to 3') Number

of bases

Forward Primer

1. SLC2A1 GGCCAAGAGTGTGCTAAAGAA 21
2. SLC2A1 Reverse Primer ACAGCGTTGATGCCAGACAG

20

1. CA2 Forward Primer GGGTACGGCAAACACAACG

19

1. CA2

Reverse Primer GGCTGTATGAGTGTCGATGTC 21

1. CUX1 Forward Primer GAAGAACCAAGCCGAAACCAT

21

1. CUX1 Reverse Primer AGGCTCTGAACCTTATGCTCA

21

07

Internal reference

08

Internal reference

GAPDH

GAPDH

Forward Primer 19

CTGGGCTACACTGAGCACC

Reverse Primer AAGTGGTCGTTGAGGGCAATG 21
