# Supplementary material for: RNA methylation-related genes of m6A, m5C, and m1A predict prognosis and immunotherapy response in cervical cancer
Source: Ann Med. 2023 Apr 12;55(1):2190618. doi: 10.1080/07853890.2023.2190618 (PMC10101678; doi:10.1080/07853890.2023.2190618)
Supplement: Supplemental Material [file IANN_A_2190618_SM7201.docx]

Supplementary Table 1. Technology Roadmap

m6A：431 related-variants

m5C: 53 related-variants

m1A：68 related-variants

From Rmavar-database

TCGA CESC RNA-seq

Tumor samples(n=232)

Normal samples(n=3)

Differential expression analysis(|logFC|>0.5 and FDR<0.05)

Go and KEGG analysis GSEA analysis

62 up -related-variants

44 down -related-variants

Univariate cox regression analysis

21 variants correlated with OS of CESC patient(p<0.1)

survive analysis

LASSO analysis

Clinicopathological characteristics

Potential drug therapy

10 variants expression signature

Tumor immune microenvironment

validation

ICI treatment

Immunohistochemistry and PCR validation of 3 potential key variants (n=30)

GSE39001

Construction and evaluation of a nomogram for prognosis prediction in TCGA cohore
